# Supplementary material for: Utility of ctDNA Liquid Biopsies from Cancer Patients: An Institutional Study of 285 ctDNA Samples
Source: Cancers (Basel). 2022 Nov 28;14(23):5859. doi: 10.3390/cancers14235859 (PMC9739663; doi:10.3390/cancers14235859)
Supplement: Supplementary file 1 [file cancers-14-05859-s001.zip › cancers-2069645-supplementary.pdf]

Supplementary Materials: Utility of ctDNA Liquid Biopsies from Cancer Patients: An Institutional Study of 285 ctDNA Samples

Josep Gumà, Karla Peña, Francesc Riu, Carmen Guilarte, Anna Hernandez, Clara Lucía, Francisca Martínez-Madueño, Maria José Miranda, Inés Cabeza, Marc Grifoll, Sergio Peralta, Sara Serrano, Félix Muñoz, Lola Delamo, Barbara Roig, Joan Borràs, Joan Badia, Marta Rodriguez-Balada and David Parada

Table S1. Mutations detected with Idylla™ Mutation EGFR, KRAS, NRAS and BRAF cartridges.

**1.1. EGFR Mutation cartridge**				
Exon 18	G719A<br>G719C<br>G719C<br>G719S	p.Gly719Ala<br>p.Gly719Cys<br>p.Gly719Cys(2)<br>p.Gly719Ser	c.2156G>C<br>c.2155G>T<br>c.2154_2155delinsTT<br>c.2155G>A	G719A/C/S
Exon 19	Del9	p.Leu747_Ala750delinsPro	c.2238_2248delinsGC	
			c.2239_2248delinsC	
		p.Leu747_Ala750delinsSer	c.2240_2248del	
		p.Leu747_Glu749del	c.2239_2247del	
	Del12	p.Leu747_Thr751delinsPro	c.2239_2251delinsC	
		p.Leu747_Thr751delinsSer	c.2240_2251del	
	Del15	p.Glu746_Ala750del	c.2235_2249del	
			c.2236_2250del	
		p.Leu747_Thr751del	c.2239_2253del	
			c.2240_2254del	
			c.2238_2252del	
		p.Glu746_Thr751delinsAla	c.2237_2251del	
		p.Glu746_Thr751delinsIle	c.2235_2252delinsAAT	
		p.Glu746_Thr751delinsVal	c.2237_2252delinsT	
		p.Lys745_Ala750delinsThr	c.2234_2248del	
		p.Glu746_Thr751delinsLeu	c.2236_2253delinsCTA	
		p.Glu746_Thr751delinsVal	c.2237_2253delinsTA	
		p.Glu746_Thr751delinsAla	c.2235_2251delinsAG	
		p.Glu746_Thr751delinsGln	c.2236_2253delinsCAA	
		p.Ile744_Ala750delinsValLys	c.2230_2249delinsGTCAA	
	Del18	p.Leu747_Pro753delinsSer	c.2240_2257del	
		p.Glu746_Ser752delinsVal	c.2237_2255delinsT	
		p.Leu747_Ser752del	c.2239_2256del	
		p.Glu746_Thr751del	c.2236_2253del	
		p.Leu747_Pro753delinsGln	c.2239_2258delinsCA	
		p.Glu746_Ser752delinsAla	c.2237_2254del	
		p.Glu746_Ser752delinsAsp	c.2238_2255del	
		p.Glu746_Ser752delinsIle	c.2236_2256delinsATC	
		p.Glu746_Ser752delinsVal	c.2237_2256delinsTT	
			c.2237_2256delinsTC	
			c.2235_2255delinsGGT	

	Del 21<br><br>Del24	p.Leu747_Pro753del<br>p.Glu746_Ser752del<br>p.Ser752_Ile759del	c.2238_2258del<br>c.2236_2256del<br>c.2253_2276del	
Exon 20	T790M<br>S768I<br>InsG<br>InsASV9<br>InsASV11<br>InsSVD<br>InsH	p.Thr790Met<br>p.Ser768Ile<br>p.Asp770_Asn771insGly<br>p.Val769_Asp770insAlaSerVal<br>p.Val769_Asp770insAlaSerVal<br>p.Asp770_Asn771insSerValAsp<br>p.His773_Val774insHis	c.2369C>T<br>c.2303G>T<br>c.2310_2311insGGT<br>c.2307_2308insGCCAGCGTG<br>c.2309_2310delinsCCAGCGTGGAT<br>c.2311_2312insGCGTGGACA<br>c.2319_2320insCAC	
Exon 21	L858R<br><br>L861Q	p.Leu858Arg<br><br>p.Leu861Gln	c.2573T>G<br>c.2573_2574delinsGT<br>c.2573_2574delinsGA<br>c.2582T>A	

**1.2. KRAS Mutation cartridge.**		
Codon12 (exon2)	G12C<br>G12R<br>G12S<br>G12A<br>G12D<br>G12V	(c.34G>T)<br>(c.34G>C)<br>(c.34G>A)<br>(c.35G>C)<br>(c.35G>A)<br>((c.38G>A)c.35G>T)
Codon 13 (exon2)	G13D	(c.38G>A)
Codon 59 (exon3)	A59E<br>A59G<br>A59T	(c.176C>A)<br>(c.176C>G)<br>(c.175G>A)
Codon 61 (exon 3)	Q61K<br>Q61L<br>Q61R<br>Q61H	(c.181C>A; c.180_181delinsAA)<br>(c.182A>T)<br>(c.182A>G)<br>(c.183A>C; c.183A>T)
Codon 117 (exon 4)	K117N	(c.351A>C; c.351A>T)
Codon 146 (exon 4)	A146P<br>A146T<br>A146V	(c.436G>C)<br>(c.436G>A)<br>(c.437C>T)

**1.3. NRAS Mutation cartridge**		
Codon12 (exon2)	G12C<br>G12S<br>G12D<br>G12A<br>G12V	(c.34G>T)<br>(c.34G>A)<br>(c.35G>A)<br>(c.35G>C)<br>(c.35G>T)
Codon 13 (exon2)	G13D<br>G13V<br>G13R	(c.38G>A)<br>(c.38G>T)<br>(c.37G>C)
Codon 59 (exon3)	A59T	(c.175G>A)
Codon 61 (exon 3)	Q61K	(c.181C>A)

	Q61L<br>Q61R<br>Q61H	(c.182A>T)<br>(c.182A>G)<br>(c.183A>C; c.183A>T)
Codon 117 (exon 4)	K117N	(c.351A>C; c.351A>T)
Codon 146 (exon 4)	A146T<br>A146V	(c.436G>A)<br>(c.437C>T)
**1.4. BRAF Mutation cartridge**		
Codon 600	BRAF V600E<br>BRAF V600E2<br>BRAF V600D<br><br>BRAF V600K<br>BRAF V600R<br>BRAF V600M<br><br>BRAF Wild Type	(c.1799T>A)<br>(c.1799_1800delinsAA)<br>(c.1799_1800delinsAT;<br>c.1799_1800delinsAC)<br><br>(c.1798_1799delinsAA)<br>(c.1798_1799delinsAG)<br>(c.1798G>A)<br><br>(c.1799T)

Table S2. Mutations detected with Idylla™ Mutation EGFR, KRAS, NRAS and BRAF cartridges.

**2.1. EGFR Mutation cartridge**		
Exon 18	G719A<br>G719C<br>G719S	c.2156G>C<br>c.2155G>T;c.2154_2155delinsTT<br>c.2155G>A
Exon 19	Del9<br><br>Del12<br><br>Del15<br><br>Del18	c.2238_2248delinsGC<br>c.2239_2248delinsC<br>c.2240_2248del<br>cc.2239_2247del<br><br>c.2239_2251delinsC<br>c.2240_2251del<br><br>c.2235_2249del<br>c.2236_2250del<br>c.2239_2253del<br>c.2240_2254del<br>c.2238_2252del<br>c.2237_2251del<br>c.2235_2252delinsAAT<br>c.2237_2252delinsT<br>c.2234_2248del<br>c.2236_2253delinsCTA<br>c.2237_2253delinsTA<br>c.2235_2251delinsAG<br>c.2236_2253delinsCAA<br>c.2230_2249delinsGTCAA<br><br>c.2240_2257del<br>c.2237_2255delinsT<br>c.2239_2256del<br>c.2236_2253del<br>c.2239_2258delinsCA<br>c.2237_2254del<br>c.2238_2255del<br>c.2237_2257delinsTCT

	Del 21<br>Del24	c.2236_2255delinsAT<br>c.2236_2256delinsATC<br>c.2237_2256delinsTT<br>c.2237_2256delinsTC<br>c.2235_2255delinsGGT<br>c.2238_2258del<br>c.2236_2256del<br>c.2253_2276del
Exon 20	T790M<br>S768I<br>InsG<br>InsASV9<br>InsASV11<br>InsSVD<br>InsH	c.2369C>T<br>c.2303G>T<br>c.2310_2311insGGT<br>c.2307_2308insGCCAGCGTG<br>c.2309_2310delinsCCAGCGTGGAT<br>c.2311_2312insGCGTGGACA<br>c.2319_2320insCAC
Exon 21	L858R<br>L861Q	c.2573T>G<br>c.2573_2574delinsGT<br>c.2573_2574delinsGA<br>c.2582T>A
**2.2. KRAS Mutation cartridge**		
Codon12 (exon2)	G12C<br>G12R<br>G12S<br>G12A<br>G12D<br>G12V	(c.34G>T)<br>(c.34G>C)<br>(c.34G>A)<br>(c.35G>C)<br>(c.35G>A)<br>(c.35G>T)
Codon 13 (exon2)	G13D	(c.38G>A)
Codon 59 (exon3)	A59E<br>A59G<br>A59T	(c.176C>A)<br>(c.176C>G)<br>(c.175G>A)
Codon 61 (exon 3)	Q61K<br>Q61L<br>Q61R<br>Q61H	(c.181C>A; c.180_181delinsAA)<br>(c.182A>T)<br>(c.182A>G)<br>(c.183A>C; c.183A>T)
Codon 117 (exon 4)	K117N	(c.351A>C; c.351A>T)
Codon 146 (exon 4)	A146P<br>A146T<br>A146V	(c.436G>C)<br>(c.436G>A)<br>(c.437C>T)

**2.3**. NRAS Mutation cartridge	
Codon12 (exon2)	(c.34G>T)<br>(c.34G>A)<br>(c.35G>A)<br>(c.35G>C)<br>(c.35G>T)

Codon 13 (exon2)	(c.38G>A)<br>(c.38G>T)<br>(c.37G>C)
Codon 59 (exon3)	(c.175G>A)
Codon 61 (exon 3)	(c.181C>A)<br>(c.182A>T)<br>(c.182A>G)<br>(c.183A>C; c.183A>T)
Codon 117 (exon 4)	(c.351A>C; c.351A>T)
Codon 146 (exon 4)	(c.436G>A)<br>(c.437C>T)
Codon12 (exon2)	(c.34G>T)<br>(c.34G>A)<br>(c.35G>A)<br>(c.35G>C)<br>(c.35G>T)
Codon 13 (exon2)	(c.38G>A)<br>(c.38G>T)<br>(c.37G>C)
Codon 59 (exon3)	(c.175G>A)
Codon 61 (exon 3)	(c.181C>A)<br>(c.182A>T)<br>(c.182A>G)<br>(c.183A>C; c.183A>T)
Codon 117 (exon 4)	(c.351A>C; c.351A>T)
Codon 146 (exon 4)	(c.436G>A)<br>(c.437C>T)

**2.4**. BRAF Mutation cartridge.		
Codon 600	BRAF V600E<br>BRAF V600E2<br>BRAF V600D<br><br>BRAF V600K<br>BRAF V600R<br>BRAF V600M<br><br>BRAF Wild Type	(c.1799T>A)<br>(c.1799_1800delinsAA)<br>(c.1799_1800delinsAT;<br>c.1799_1800delinsAC)<br><br>(c.1798_1799delinsAA)<br>(c.1798_1799delinsAG)<br>(c.1798G>A)<br><br>(c.1799T)
